# Validating brain activity measures as reliable indicators of individual diagnostic group and genetically mediated sub-group membership Fragile X Syndrome

**DOI:** 10.21203/rs.3.rs-3849272/v1

**Published:** 2024-01-18

**Authors:** Lauren E. Ethridge, Ernest V. Pedapati, Lauren M. Schmitt, Jordan E. Norris, Emma Auger, Lisa A. De Stefano, John A. Sweeney, Craig A. Erickson

**Affiliations:** University of Oklahoma; Cincinnati Children’s Hospital Medical Center; Cincinnati Children’s Hospital Medical Center; University of Oklahoma; University of Oklahoma; Cincinnati Children’s Hospital Medical Center; University of Cincinnati College of Medicine; Cincinnati Children’s Hospital Medical Center

## Abstract

Recent failures translating preclinical behavioral treatment effects to positive clinical trial results in humans with Fragile X Syndrome (FXS) support refocusing attention on biological pathways and associated measures, such as electroencephalography (EEG), with strong translational potential and small molecule target engagement. This study utilized guided machine learning to test promising translational EEG measures (resting power and auditory chirp oscillatory variables) in a large heterogeneous sample of individuals with FXS to identify best performing EEG variables for reliably separating individuals with FXS, and genetically-mediated subgroups within FXS, from typically developing controls. Best performing variables included resting relative frontal theta power, all combined whole-head resting power bands, posterior peak alpha frequency (PAF), combined PAF across all measured regions, combined theta, alpha, and gamma power during the chirp, and all combined chirp oscillatory variables. Sub-group analyses best discriminated non-mosaic FXS males via whole-head resting relative power (AUC = .9250), even with data reduced to a 20-channel clinical montage. FXS females were nearly perfectly discriminated by combined theta, alpha, and gamma power during the chirp (AUC = .9522). Results support use of resting and auditory oscillatory tasks to reliably identify neural deficit in FXS, and to identify specific translational targets for genetically-mediated sub-groups, supporting potential points for stratification.

## 1. Introduction

Fragile X Syndrome (FXS) is a neurodevelopmental disorder caused by a trinucleotide repeat expansion of more than 200 repeats in the promoter region of the Fragile X messenger ribonucleoprotein 1 (*FMR1*) gene resulting in gene methylation and partial or complete transcriptional silencing. FXS is clinically characterized by intellectual disability, social anxiety, autistic features, and sensory sensitivities. Due to the location of the *FMR1* gene on the X chromosome, males are typically more severely affected than females, although females may experience the full range of symptoms, including significant intellectual disability.

Considerable effort has gone into small molecule target treatment development specific to FXS, with limited success. Recent clinical trial failures have highlighted several possible reasons for the lack of successful Phase 3 trials in FXS, including sample heterogeneity and the use of caregiver-report outcome measures that may rely too heavily on subjective response and may show strong placebo responses [1, 2]. Additionally, the difficulties in replicating the strong treatment effects of mGlur5 antagonists found in preclinical models to human trials [3, 4] has implicated the need for a refocusing of attention to biological pathways and measures that show strong translational potential as treatment targets across species. One methodology that may meet these criteria is electroencephalography (EEG) [5]. Specifically, our research group and others have shown that abnormalities in resting spectral power, particularly in the theta, alpha, and gamma bands, translate strongly across *FMR1* KO mouse and humans with FXS and may be responsive to treatment [6–12]. Additionally, sensory abnormalities measured via EEG including neural oscillatory synchronization to an auditory chirp stimulus are conserved across species [13] [14] and show small molecule target engagement in *FMR1* KO mice [8, 15–16]. Further, preclinical models have highlighted specific cell types [17, 18]and brain structures [19] that may underlie these auditory chirp oscillatory abnormalities, and thus may serve as good targets for pharmacological intervention. Additional preclinical work has suggested that these phenotypes are specific to the auditory modality [20], further narrowing the field of inquiry to manageable outcomes. Of interest, many of the novel pharmaceuticals that have showed improvements in the chirp oscillatory phenotype in *FMR1* KO mice are not targeted directly at the mGluR theory of FXS, but rather at alternative pathways such as endocannabinoid effects [16] and PDE10A inhibition [15]. Recent human trials of cannabidiol gel [21, 22] and PDE4D inhibition [23] also have shown promise, including marginal effects of the latter on related auditory EEG biomarkers.

In identifying the pathways associated with specific deficits in FXS, it is also important to resolve heterogeneity across individuals, not only to facilitate potential clinical trial stratification to improve the likelihood of interpretable outcomes, but also to effectively match treatments to those most likely to benefit (i.e. personalized medicine). For example, certain EEG phenotypes and their associated biological pathways may be more affected in individuals who express the least Fragile X messenger ribonucleoprotein (FMRP), i.e., fully methylated, non-mosaic FXS males. Finally, any EEG phenotypes that are present in nearly all individuals with FXS and do not show strong effects of sex or mosaicism may successfully serve as outcome measures for trials with more heterogenous samples. In this study, we utilized guided machine learning to identify which of the most promising translational EEG measures, including resting power variables and auditory chirp oscillatory variables, best differentiate individuals with FXS and genetically mediated subgroups within FXS from typically developing controls (CON), using a large, heterogeneous sample. We predicted that chirp would more reliably separate individuals with FXS from CON due to the expected reduction in individual neural variability associated with stimulus-evoked tasks over resting state EEG, and that composites consisting of multiple variables from the same task would be more robust identifiers than any single variable due to the enhancement in signal to noise in using multiple measures of neural activity.

## 2. Results

### 2.1 Resting EEG.

**Figure 1:**
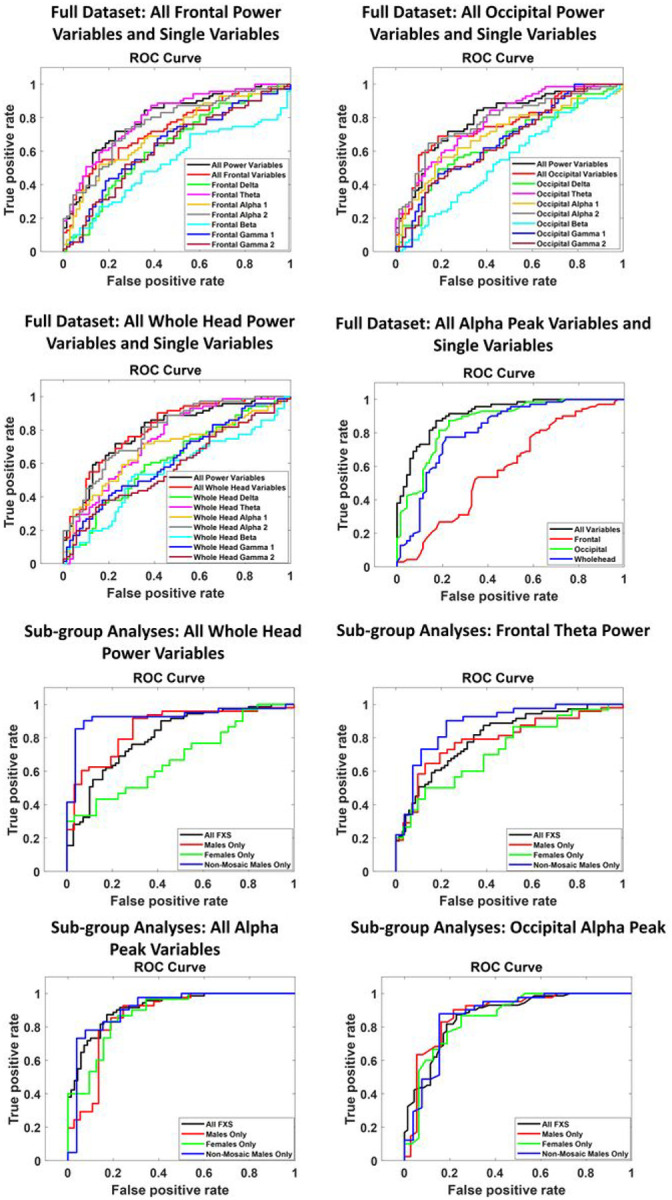
Top four panels. Receiver operating characteristic (ROC) curves for all resting EEG variables and variable combinations tested across the entire dataset. Bottom four panels. ROC curves for top performing resting EEG variables/variable combinations for the entire dataset, applied to separation of FXS males, FXS females, and FXS non-mosaic males vs their matched typically developing counterparts. The black line in each sub-group plot replicates the performance of the variables for the entire dataset, for comparison.

**Figure 2:**
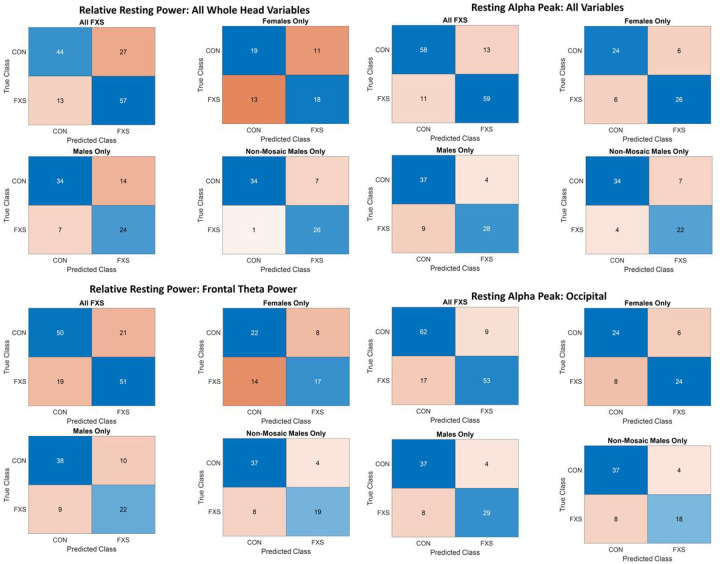
Confusion matrices for each of the four best performing resting EEG variables/variable combinations, separated by entire dataset (all FXS), FXS males, FXS females, and FXS non-mosaic males vs their matched typically developing counterparts. Blue boxes indicate correct predictions, while red boxes indicate incorrect predictions, with darker shading associated with relatively larger values in each box compared to total.

Receiver operating characteristic (ROC) curves and confusion matrices are presented for the top performing variable sets for the entire dataset and their subsequent sub-group analyses in Figures 1 and 2, respectively. All model results are presented in Table 1 for absolute power, Table 2 for relative power, and Table 3 for alpha peak measures. Performance was assessed using area under the curve (AUC) and cross-validation classification error. For absolute power, no variable or predefined combination of variables outperformed the two best performing measures for relative power and peak alpha frequency (PAF), therefore results at the diagnostic group level were not pursued at the subgroup level for absolute power. The likely reason for underperformance in absolute power is increased individual variability (Table 4). For relative power, the best performing measures, were frontal theta (AUC=.8050; error=.2766), driven by a relative increase in theta for FXS over CON (Table 5) and a combination of all power variables averaged over the whole head (AUC=.8066, error=.2908), driven by a more complex interaction between increased low and high frequency power in FXS but decreased alpha power relative to CON (Table 5). These two variables were further analyzed for improvement in classification for genetically defined sub-groups: all males, all females, and non-mosaic males. Diagnostic group prediction accuracy was worse for both frontal theta and all power variables combined across the whole head for females, whereas the whole head composite performed slightly better for males and reached the outstanding range (greater than .90; [24,25]) for classifying non-mosaic FXS males vs. CON males (AUC=.9250, error=.1765).

For alpha peak, the two best performing measures were posterior alpha peak (AUC=.8686, error=.2057), in the excellent range, driven by a decrease in alpha peak values in FXS relative to CON (Table 6) and a combination of alpha peak values for frontal, posterior, and whole head regions (AUC=.9161, error=.1986), in the outstanding range. This combination reflected a similar to slightly increased frontal alpha peak in FXS, but decrease frontal and whole head alpha peak values relative to CON. Diagnostic group prediction accuracy remained similar across sub-group analyses, including the most homogeneous sub-group of non-mosaic FXS males (AUC=.9118, error=.1940), consistent with the relatively stable alpha peak values across these sub-groups.

#### 2.1.1 Data reduction for 20-channel montage.

Best-performing resting variable sets were re-computed for prediction accuracy on the best-performing group analysis using a down-sampled 20-channel montage to assess the scalability of dense array solutions to simpler clinical EEG applications. This was not performed for chirp variables, as these measures reflect a small cluster of channels rather than whole-head measurements. For relative resting power, the best performing metric for the 128-channel solution was all whole head variables for the non-mosaic FXS males. When reduced to a 20-channel clinical montage, the performance for this variable set remains in the outstanding range (AUC=.9070, error=.2206), indicating robustness of this measure to scalable data collection options. For alpha peak, the best-performing metric for the 128-channel solution was all variables for the entire FXS diagnostic group. The outstanding performance for this variable set did not survive reduction to a 20-channel montage (AUC=.6592, error=.3901).

### 2.2 Chirp EEG.

**Figure 3:**
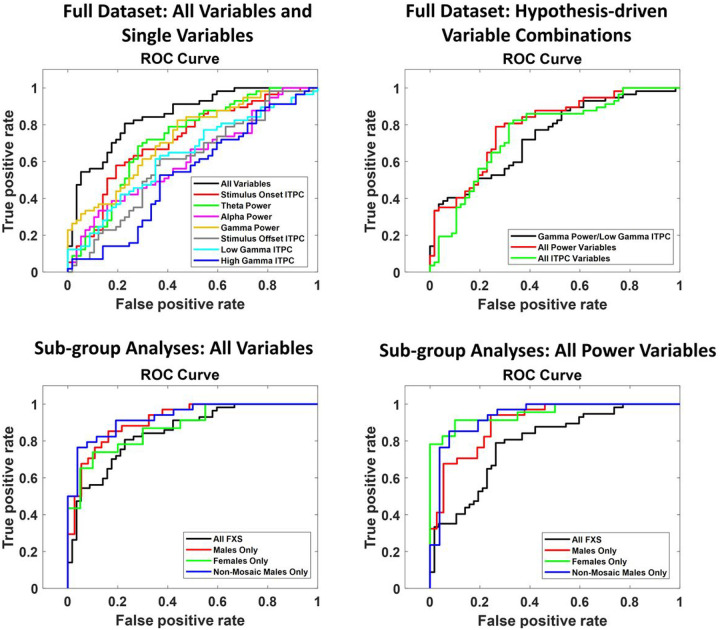
Top panels. Receiver operating characteristic (ROC) curves for all chirp EEG variables and variable combinations tested across the entire dataset. Bottom panels. ROC curves for top performing chirp EEG variables/variable combinations for the entire dataset, applied to separation of FXS males, FXS females, and FXS non-mosaic males vs their matched typically developing counterparts. The black line in each sub-group plot replicates the performance of the variables for the entire dataset, for comparison.

**Figure 4:**
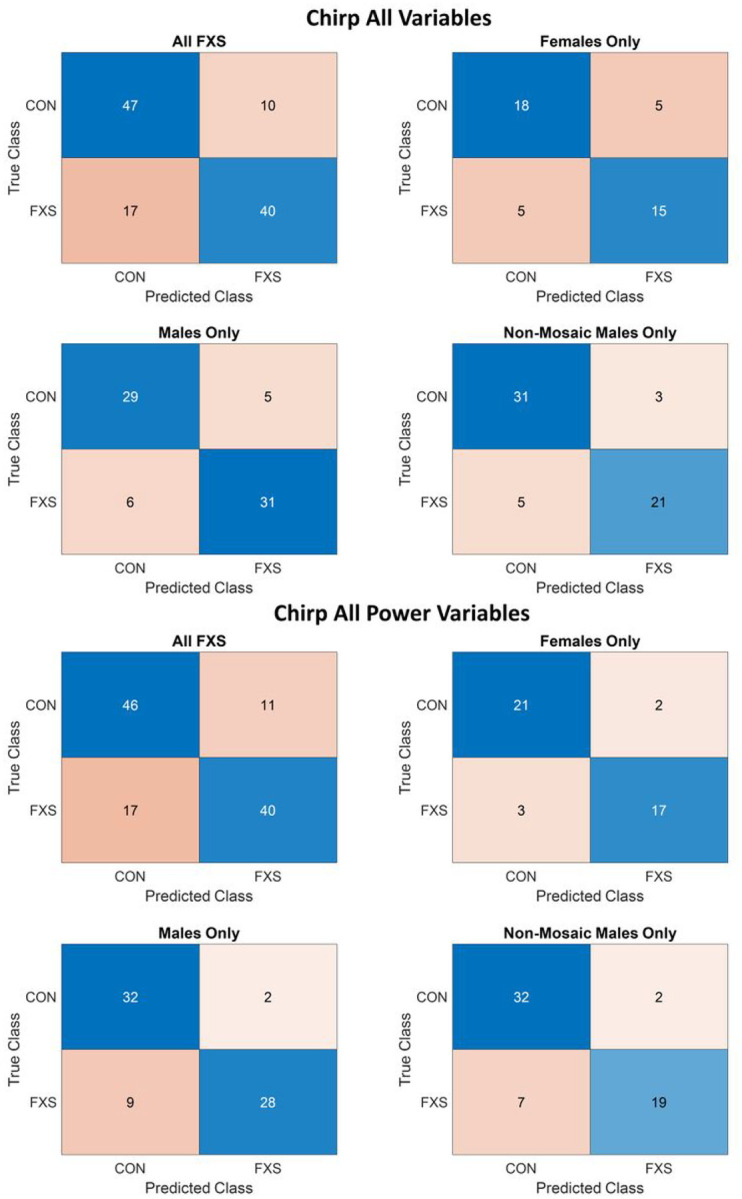
Confusion matrices for the two best performing chirp EEG variables/variable combinations, separated by entire dataset (all FXS), FXS males, FXS females, and FXS non-mosaic males vs their matched typically developing counterparts. Blue boxes indicate correct predictions, while red boxes indicate incorrect predictions, with darker shading associated with relatively larger values in each box compared to total.

**Figure 5:**
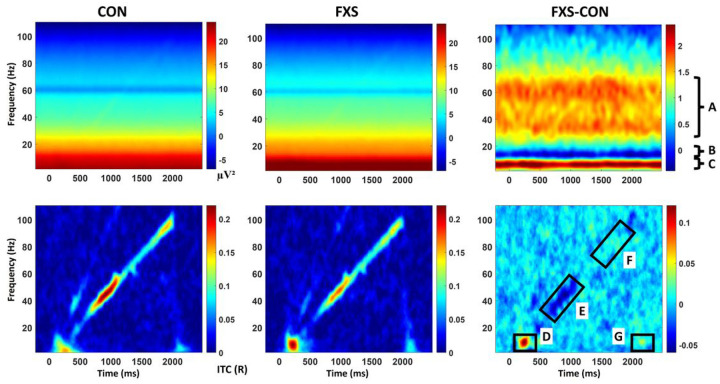
Time frequency plots of chirp STP (top panels) and ITPC (bottom panels) for typically developing controls (CON) and FXS. Difference plots for each measure indicate with black brackets (top; brackets indicate the frequency range for each variable, measured across the entire time range with the exception of evoked alpha power measured post-baseline) and boxes (bottom) the variables of interest selected a priori for analysis. A) gamma power, B) evoked alpha power, C) theta power, D) theta/alpha ITPC to the stimulus onset, E) low gamma (centered on 40 Hz) ITPC to the chirp stimulus, F) high gamma (centered on 80 Hz) ITPC to the chirp stimulus, G) theta/alpha ITPC to the stimulus offset.

ROC curves and confusion matrices are presented for the top performing variable sets for the entire dataset and their subsequent sub-group analyses in Figures 3 and 4, respectively. All model results are presented in Table 7. The two best performing measures for the chirp EEG task were the combination of all power variables (AUC=.7907, error=.2807), in the good range, driven by increases in theta and gamma power in FXS relative to CON, with a large decrease from baseline in alpha power in FXS relative to CON, and the combination or all variables (AUC=.8496, error=.2982), in the excellent range, which reflected the above differences plus a decrease in low gamma ITPC and increase in low frequency ITPC to stimulus onset in FXS (Table 6). For both variable sets, sub-grouping increased performance into the outstanding range for males and non-mosaic males, however, the highest AUC for sub-group analyses for these two variable sets was found for all power variables in FXS females (AUC=.9522, error=.3256), equating to misclassification of only 2 CON and 3 FXS participants (Fig 5).

## 3. Discussion

In this study, we aimed to utilize guided machine learning to test the most promising translational EEG measures (resting EEG power, and auditory chirp oscillatory variables) in a large heterogeneous sample of individuals with FXS, to address the sensitivity and specificity at correctly grouping individuals for EEG measures that consistently separate FXS from CON at the group-level [6, 7, 9, 12, 26]. In developing and validating a biomarker for use in clinical applications, the biomarker must map on to a particular mechanistic or biologic process, it must show reliability across multiple testing, and it must be able to provide information about participants at the individual level rather than via group statistics [27]. Although definitive mechanistic explanations for resting and chirp EEG abnormalities in FXS have not been formalized, the translational nature of these measures to preclinical rodent models has provided the background in which to do so [10, 11, 13, 14, 28]. In fact, a number of studies in the *fmr1* KO mouse both in vivo and in vitro have narrowed the focus for circuit and molecular level understanding of biologic contributors to both resting and chirp EEG findings in FXS. In particular, studies in which *fmr1* was conditionally knocked-out in excitatory cells in forebrain cortex [17] and inferior colliculus [19] have begun to dissociate features of the chirp EEG to subcortically-mediated synchronization deficits that are then inherited by auditory cortex from cortically-driven gamma power enhancements. Cortically mediated gamma power enhancement is also supported mechanistically by findings of enhanced synchronization between layers 2/3 and 5 in auditory cortex, indicating that local cortical circuits are hyperexcitable in the gamma frequency range [29], whereas subcortical structures drive flexibility to changing task demands such as those presented by the linearly changing synchronization frequency in the chirp task [19]. Resting power abnormalities in FXS may reflect similar mechanisms for gamma power enhancement [16, 30] or may reflect thalamic or hippocampal inhibitory deficits via changes to cross-frequency coupling between theta, alpha and gamma power [9, 12, 31]. Reliability across multiple testing remains an open question, although preliminary work suggests moderate to strong reliability for resting EEG in a wide range of individuals with FXS [32] and moderate reliability for gamma power during auditory tasks, at least in young individuals with FXS [33]. In this study, we utilized guided machine learning to delineate individual level predictions for diagnostic group and subgroup membership (FXS or CON, sex and mosaicism status), to address the third requirement for an effective biomarker.

We predicted that chirp EEG variables would be more successful at reliably differentiating FXS from CON, and that multiple variables composites from the same task would enhance signal-to-noise ratio in neural data and thus be more robust identifiers than any single variable. In slight contrast to our prediction, best-performing variables across chirp and resting EEG performed equally well overall, with the best-performing variables (in each case a composite) classifying FXS from CON with AUC values in the excellent (> .80) range for both tasks. Of note, classification strength from an algorithm based on Bayes rules, such as was used here, does not directly correspond to prediction strength with frequentist statistics (i.e., there is not a linear relationship between prediction strength and statistical magnitude). Therefore it is entirely possible to find larger group separation effects for the chirp task statistically while classification of individuals can be performed equally well for both chirp and resting tasks.

The overall best-performing measure for classifying all individuals with FXS was a composite of alpha peak across frontal, posterior, and whole head areas, with an AUC in the excellent (> .90) range. Performance did not noticeably increase for alpha peak measures when applied to more homogeneous sub-groups, suggesting that alpha peak is generally affected across all individuals with FXS, regardless of sex or mosaicism status. This finding is consistent with recent research showing a general slowing of the alpha peak frequency for both males and females with FXS [9] (but see [26]or an example of an intermediate PAF slowing effect in females with FXS).

Sub-group analyses showed more differentiated performance for the remaining EEG tasks and measures. For resting EEG, both frontal theta and all whole-head relative power variables performed relatively poorly for classifying females with FXS from female CON, but performed better for classifying males with FXS and non-mosaic males with FXS from male CON, suggesting that relative power abnormalities are more consistent in individuals with FXS with the least FMRP [34]. Conversely, all power variables, which consisted of absolute power and task-evoked power measures rather than relative power, performed the best at classifying females with FXS for the chirp task, with the highest AUC value over all comparisons (AUC = .9520). The cross-validation error term for this measure was somewhat increased relative to those for other best-performing sub-group analyses, however, which may be reflective of the smaller sample size for females with FXS and must be interpreted with caution. Regardless, absolute power, particularly in the frontal region in which the chirp data was measured, has been recently found to differ in dynamic utilization and timing between males and females with FXS, and may reflect different underlying mechanistic and compensatory processes [35].

Importantly, all best performing variables for classifying diagnostic groups consisted of combinations of variables rather than any one single variable. One simple explanation for this finding may be the enhancement in signal to noise when using multiple measures that reflect similar pathophysiology. A more plausible explanation may be that any one measure is not reliably different in FXS; rather it is the pattern of changes in EEG across multiple measures that is inherent to the pathophysiology of FXS. One might expect similar concerns when classifying FXS from other conditions like autism spectrum disorder [36], and schizophrenia [37], where some EEG phenotypes may overlap, but the overall pattern of EEG changes across all measures is specific to each diagnostic group. Because the machine learning algorithm used here, the naive Bayes classifier, evaluates equal contributions from linear combinations of input variables, solutions from this classification strategy readily translate to single linear composite variables that can then be used for clinical correlations and to track response to treatment.

One critical point of discussion is the application for which a particular biomarker will be used clinically. In the case of FXS, a genetic test is definitive for diagnostic purposes, and to classify molecular genetic mosaicism-status, therefore EEG would be considered redundant and more time-consuming for these purposes even with perfect classification performance. The purpose of delineating diagnostic groups and sub-groups for this study was not to replace current gold-standard diagnostic testing, but to determine which EEG phenotypes best characterize the FXS brain at the individual level. In this sense, a reliable marker that is mechanistically linked to specific biologic processes may be more useful than broad behavioral testing or clinical global impression scales in quantifying target engagement and biologic consequences for novel treatments, given that it is individually present in a specific diagnostic group or sub-group, for examples non-mosaic males but not mosaic males or females with FXS. Better or more robust treatments may emerge from efforts to differentiate the EEG phenotype of FXS from other NDDs using similar methods as well.

While the findings of this study are robust, given the use of the NBC, and have important implications for the FXS field, this study had some limitations. Although the naïve Bayes classifier is very robust to smaller sample size, some of the sub-groups samples, in particular non-mosaic males, were relatively small compared to the overall sample. However, the cross-validation error was generally robust for the non-mosaic male sub-group on best performing variables, somewhat mitigating this concern. Secondly, ideally in a biomarker validation study retest reliability would also be considered, however retest data was not available for all individuals (but see [32]) and is thus an ongoing pursuit for future study. Third, we only analyzed classification accuracy within task, and did not evaluate combined accuracy across rest and chirp tasks. This was done deliberately to determine whether one task was inherently better performing, thus reducing participant burden during data collection. Our findings indicate that neither task is clearly superior to the other in terms of classification performance, and thus the recommendation is to utilize the task most fitting to the clinical question and targeted sub-group. Both tasks performed very well at classifying individuals when composite variables were evaluated, indicating that including multiple tasks in an EEG battery as a matter of course simply to increase accuracy may involve diminishing returns. One counterpoint to this recommendation, however, is the short amount of time (5 minutes or less) that was required to collect the eyes open resting EEG data used in this study, which may not represent sufficient clinical burden to justify removal.

Group-level comparisons and robust translational work have provided a strong background for considering resting EEG and auditory stimulus-evoked EEG tasks such as the chirp task as candidate biomarkers for disruptions to neural processes in FXS [6, 9]. This study marks the first effort to delineate the ability of these biomarkers to *individually* characterize and classify the FXS brain, a required step in biomarker validation. We found that both resting and chirp EEG robustly classify individuals by diagnostic group and genetically mediated sub-group, showing both sensitivity and specificity against typically developing controls. Future research will determine specificity against similar conditions with overlapping phenotypes (i.e., other NDDs, like idiopathic autism spectrum disorder), however the use of composite variables that describe patterns of brain activity, rather than single measures in isolation, increases likelihood of specificity to FXS. The current sample was heterogeneous, reflecting a wide range of clinical severity, age, medication use, and included both sexes, yet composite EEG variables from both tasks performed well in classifying individuals despite these hurdles. This robust performance suggests that the neural composites found here are specific to FXS and not to a research domain criterion-type concept such as intellectual/cognitive ability or autistic features. The differences in sub-group performance across measures may reflect the more sensory focus of the chirp task compared to the more general resting EEG, and thus one task may be more appropriate than another for particular clinical trial applications, depending on the pharmacological target. The breadth of preclinical work using this highly translational phenotypes, including ideally preclinical work focused on composite variables, will continue to further refine the unique mechanisms contributing to individual differences in these phenotypes, and the overlapping mechanisms inherent to neural phenotypes in FXS as a whole. These simple, short EEG composites represent feasible tasks that reflect a range of clinical characteristics in FXS [6, 7, 9, 26], are a reliable indicator of individual deficit, and are robust to noise from single variables, supporting their use in the development and outcome testing of novel drug targets with increased chance of clinical relevance in randomized controlled trials.

## 4. Methods

### 4.1 Participants.

Participants with FXS had a genetic diagnosis confirmed via Southern Blot and polymerase chain reaction (PCR). Exclusion criteria were present history of seizures within one year and current regular use of benzodiazepines. Typically developing controls additionally were excluded if they were receiving current treatment for neuropsychiatric illness as ascertained via clinical interview. All participants provided written informed consent, or assent as appropriate prior to participation. Study activities were approved by the institutional review boards at Cincinnati Children’s Hospital Medical Center and University of Oklahoma, as appropriate, following ethical guidelines in accordance with the Declaration of Helsinki.

Participants for the resting EEG dataset included 70 individuals with a genetic diagnosis of FXS (mean age = 20.5, SD = 10; age range = 5.9–45.7; 54% male) and 71 typically developing controls (mean age = 22.2, SD = 10.7; age range = 5.9–48.2; 57% male). Of the 38 males with FXS, 12 had methylation or size mosaicism. This sample has been previously described in [9]. Participants for the chirp dataset included 57 individuals with FXS (mean age = 24.51; SD = 9.64; age range = 9.1–45.7 years; 65% male) and 57 typically developing controls (mean age = 24.64; SD = 12.30; age range = 8.1–57.1 years; 60% male). Of the 37 males with FXS, 9 had methylation mosaicism. The chirp sample includes data from [6] plus 40 (22 FXS and 18 CON) additional, newer data collected under the same parameters.

As is common in FXS populations, many FXS participants were taking concurrent medications. Individuals taking concurrent medications, including antidepressants and antipsychotics, were required to be on a stable dose for at least six weeks prior to participation. Removing participants for concurrent medication usage beyond those with known EEG effects (i.e. benzodiazepines) risks creating a non-representative sample of individuals with no severe symptoms. As the goal of the study was to identify EEG biomarkers that are robust to heterogeneity in clinical samples and/or sensitive to specific subgroups, including those on concurrent medications was considered the most representative of actual participant samples that would be recruited for clinical trials. For the resting EEG dataset, 35 FXS participants were taking antidepressants,18 were taking atypical antipsychotics, and 20 were taking stimulants. For the chirp EEG dataset, 23 FXS participants were taking antidepressants,16 were taking atypical antipsychotics, and 5 were taking stimulants.

### 4.2 EEG Data and Variable Selection.

EEG data acquisition has been described in detail for these tasks in [6] and [9]. Briefly, EEG data was collected continuously using 128-channel EGI Hydrocel nets (EGI/MagStim, Eugene, OR) while participants sat quietly for 5 minutes watching a standardized silent video (rest) and while participants listened passively to auditory chirp stimulus consisting of broadband noise amplitude modulated by a sinusoid that increased linearly from 0–100 Hz modulation frequency over 2 seconds. Chirp trials were presented 200 times with an inter-stimulus interval randomly jittered between 1500–2000 ms.

Details on variable derivation are described in [6] and [9]. Briefly, artifacts were removed using independent components analysis. Absolute (raw amplitude) and relative (amplitude within a band corrected for total power) were derived using Fast Fourier transform over data segmented into 2 second epochs, with 0.5 Hz frequency steps. Alpha peak frequency was calculated as the peak of the maximum absolute log power between 6–14 Hz. Chirp variables, single trial power (STP) and inter-trial phase coherence (ITPC) were calculated in the time-frequency domain using Morlet wavelets with 1 Hz frequency step linearly increasing from 1–30 cycles. Inter-trial phase coherence is the consistency of the phase of a particular waveform over multiple trials, and single trial power is the amplitude of the waveform across trials irrespective of phase.

For group classification analysis, resting and chirp tasks were analyzed separately, to provide comparative performance between tasks, rather than combining variables across tasks. For each task, initial variables were selected based on previous work indicating significant differences between FXS and CON in at least one analysis. For resting EEG, variable selection was performed based on findings from [9] and [12], including all power bands for completeness. The resting dataset used in this study is the same dataset reported in [9]. For chirp EEG, variable selection was performed based on findings from [7] and [6]. The chirp dataset partially overlaps with the dataset reported in [6]. Since the purpose of this study was to find the most useful outcome measures, which includes consideration of feasibility, although source analyzed variables are also reported in [9], we opted to retain EEG data in both tasks from the scalp only, which may be more easily collected in clinical trial settings. Final variables for resting EEG were absolute and relative power in all power bands (delta, theta, low alpha, high alpha, beta, low gamma, high gamma), as well as alpha peak frequency, calculated across the frontal and posterior quadrants of the scalp (see [9] for sensors used) as well as averaged across the whole head for a total of 45 variables. Final variables for chirp EEG were theta/alpha ITPC to the stimulus onset, low gamma (centered on 40 Hz) ITPC to the chirp stimulus, high gamma (centered on 80 Hz) ITPC to the chirp stimulus, theta/alpha ITPC to the stimulus offset, theta absolute STP, gamma absolute STP, evoked (baseline-corrected) absolute alpha STP for a total of 7 variables. All chirp variables were calculated over a fronto-central scalp distribution of sensors consistent with neural activity projected from auditory cortices. See Fig. 5 and [6] for more detail on variable definitions.

For each task, variables were each tested individually for group separation using a machine learning classifier (see section 4.3 for details on the classifier). Then, the combination of all variables within a task were tested together, with the exception that for resting EEG, variables for absolute power, relative power, and alpha peak were analyzed as separate combinations rather than combining all 45 resting variables into a single analysis, due to the reduced interpretability of such a high dimensional dataset. To reduce the effect of multiple comparisons, not all possible combinations of variables were tested; rather, hypothesis-driven combinations were tested based on previous work. Specific combinations for resting EEG were all frontal variables, all posterior variables, and all whole-head variables. Specific combinations for chirp EEG were all power variables, all ITPC variables, and a combination of gamma power and low gamma ITPC. Generally, we expected frontal variables and power variables to outperform other areas and ITPC, but that the specific combination of gamma power and low gamma ITPC, which have shown an inverse relationship and consistent abnormalities in FXS [7] [6], would perform well compared to either single variable.

The best performing variables or combinations of variables at separating all FXS individuals from all CON individuals were then re-trained and tested on genetically mediated subgroups of FXS to determine whether creating more homogeneous groups improved performance on these already well-performing solutions. Specifically, we evaluated sex effect by testing group separation for all male FXS vs. all male CON participants, and all female FXS vs. all female CON participants. Then we tested for effects of methylation mosaicism status as a proxy for amount of protein produced (i.e., generally the most severely affected will have little or no protein) by testing the non-mosaic, fully methylated male FXS vs all male CON participants. Additionally, as one of the core purposes of this study was to identify best performing variables for clinical trial usage, and all data were collected using a 128-channel dense-array EEG system, which may not be feasible in many clinical trial settings, we further tested best performing resting EEG variables by down-sampling data to a 20-channel standard 10–20 montage and recomputing prediction metrics to indicate scalability of machine learning outcomes to simplified EEG set-ups. Chirp data already represents the average of a small cluster of 23-channels on fronto-central scalp, therefore we consider this measure to be relatively scalable as originally computed.

### 4.3 Machine Learning Analyses.

Selected variables were input into a naïve Bayes classifier machine learning algorithm to assess performance on separating FXS from CON. The naïve Bayes classifier (NBC), implemented in Matlab R2021b, was chosen specifically due to its robust performance in relatively small sample sizes, and the assumption that each variable contributes linearly and equally to the classifier solution. Although the sample of FXS and CON used in this study is large relative to other studies in single gene neurodevelopmental disorders, some machine learning algorithms recommend 150 to thousands of observations to achieve good model performance, while the independence assumption inherent to the naïve Bayes classifier allows it to learn high dimensional features and achieve model fit with much smaller training sets [38, 39]. Additionally, while nonlinear solutions may ultimately provide a better model fit and may successfully classify more individuals, the purpose of this study was to identify variables or combinations of variables that are most useful as outcome measures for target engagement or for stratification in preclinical work and/or clinical trials. Nonlinear solutions are often difficult to interpret or translate directly to single outcome measures, and may present problems for identifying straightforward cutoffs for participant stratification. Linear solutions provide more interpretable boundaries between groups, and lend themselves easily to creation of composite variables, thus supporting the naïve Bayes classifier as the best candidate for the applied purposes of this study.

For each variable or set of variables, 70% of the participants (evenly distributed across FXS and CON) were randomly selected for training the NBC, with the remaining 30% withheld for testing the model accuracy and generalizability. Priors were set at 50% for each group as group sizes were approximately equal. Classification results were quantified using the area under the curve (AUC) for the receiver operating criteria (ROC) curve and are reported for both the 30% testing set and for the model applied back to the entire dataset. The ROC curve is created by plotting the false positive rate against the true positive rate, with AUC values closer to 1 indicating better classification performance. To avoid misrepresenting performance in small subgroups (e.g., females) due to random selection of the testing set, 10-fold cross-validation was also used to compute the cross-validation error for each solution.

## Figures and Tables

**Table 1.** Classification Results for Resting Absolute Power

Variable	AUC – 30% holdout test set	AUC – entire dataset	Cross Validation Classification Error
**ALL SUBJECTS**			
All Frontal Variables	.4830	.5604	.4397
Frontal Delta	.5669	.5421	.4610
Frontal Theta	.6644	.6920	.4043
Frontal Alpha 1	.4898	.5543	.5319
Frontal Alpha 2	.4807	.4559	.4823
Frontal Beta	.5465	.5274	.4468
Frontal Gamma 1	.5465	.5449	.4255
Frontal Gamma 2	.6689	.5400	.4184
All Posterior Variables	.6848	.6710	.4113
Posterior Delta	.6508	.6704	.4184
Posterior Theta	.6372	.7103	.4113
Posterior Alpha 1	.5737	.5753	.4610
Posterior Alpha 2	.5125	.5795	.5035
Posterior Beta	.5873	.6282	.4184
Posterior Gamma 1	.6304	.6813	.4113
Posterior Gamma 2	.7506	.6763	.4043
All Whole Head Variables	.7642	.7308	.3617
Whole Head Delta	.5374	.5445	.4255
Whole Head Theta	.6576	.6469	.4255
Whole Head Alpha 1	.4943	.5654	.4823
Whole Head Alpha 2	.6803	.6020	.4894
Whole Head Beta	.4490	.6000	.4043
Whole Head Gamma 1	.8322	.7187	.3262
Whole Head Gamma 2	.7483	.7139	.3546
All Variables	.5510	.6906	.3901

**Table 2.** Classification Results for Resting Relative Power

Variable	AUC – 30% holdout test set	AUC – entire dataset	Cross Validation Classification Error
**ALL SUBJECTS**			
All Frontal Variables	.5215	.7384	.3121
Frontal Delta	.5737	.6308	.4255
Frontal Theta	.6599	.8050	.2766
Frontal Alpha 1	.6936	.7093	.3617
Frontal Alpha 2	.7868	.7656	.3475
Frontal Beta	.5329	.5280	.4681
Frontal Gamma 1	.6054	.6352	.3972
Frontal Gamma 2	.5125	.6239	.4184
All Posterior Variables	.8707	.7598	.2553
Posterior Delta	.6735	.6553	.4043
Posterior Theta	.6984	.7827	.3121
Posterior Alpha 1	.6259	.7012	.3475
Posterior Alpha 2	.8458	.7801	.2695
Posterior Beta	.6236	.5459	.4397
Posterior Gamma 1	.7642	.6646	.4043
Posterior Gamma 2	.7029	.6565	.4043
All Whole Head Variables	.8005	.8066	.2908
Whole Head Delta	.5805	.6193	.4113
Whole Head Theta	.8005	.7410	.2979
Whole Head Alpha 1	.6190	.6867	.3617
Whole Head Alpha 2	.7868	.7911	.3121
Whole Head Beta	.5170	.5590	.5035
Whole Head Gamma 1	.6961	.6260	.4539
Whole Head Gamma 2	.5669	.5821	.5035
All Variables	.7120	.7956	.2695
**SUB-GROUP ANALYSES**			
**MALES ONLY**			
All Whole Head Variables	.7619	.8575	.3165
Frontal Theta	.7381	.7870	.2658
**FEMALES ONLY**			
All Whole Head Variables	.6914	.6785	.4262
Frontal Theta	.8642	.7247	.3607
**NON-MOSAIC MALES ONLY**			
All Whole Head Variables	.9375	.9250	.1765
Frontal Theta	.8750	.8799	.1471
**NON-MOSAIC MALES ONLY: 20 CHANNEL MONTAGE**			
All Whole Head Variables	.7917	.9070	.2206

Note: Top 2 highest performing variables for all subjects are highlighted in grey. Sub-group analyses for males, females, and non-mosaic males reflect these 2 variables only. 20 channel montage analyses reflect only the highest performing variable and sub-group from the sub-group analyses.

**Table 3.** Classification Results for Resting Alpha Peak

Variable	AUC – 30% holdout test set	AUC – entire dataset	Cross Validation Classification Error
**ALL SUBJECTS**			
Frontal	.5748	.6272	.4468
Posterior	.9218	.8686	.2057
Whole Head	.9229	.8147	.2837
All Variables	.9569	.9161	.1986
**SUB-GROUP ANALYSES**			
**MALES ONLY**			
All Variables	.7803	.8675	.1795
Posterior	.9545	.8817	.1795
**FEMALES ONLY**			
All Variables	.9136	.8823	.2258
Posterior	.8765	.8573	.2419
**NON-MOSAIC MALES ONLY**			
All Variables	.8542	.9118	.1940
Posterior	.8854	.8696	.1791
**ALL SUBJECTS: 20 CHANNEL MONTAGE**			
All Variables	.7778	.6592	.3901

Note: Top 2 highest performing variables for all subjects are highlighted in grey. Sub-group analyses for males, females, and non-mosaic males reflect these 2 variables only. 20 channel montage analyses reflect only the highest performing variable and sub-group from the sub-group analyses.

**Table 4:** Means for Absolute Resting Power by Group and Sub-Group

Variable	All FXS	All CON	FXS males	FXS females	FXS non-mosaic males	CON males	CON females
**N**	70	71	38	32	27	41	30
Frontal Delta	3.45(2.96)	2.15(1.74)	4.13(3.44)	2.65(2.04)	4.30(3.56)	2.48(1.95)	1.70(1.32)
Frontal Theta	2.63(2.12)	1.44(1.24)	3.11(2.37)	2.05(1.65)	3.42(2.59)	1.58(1.28)	1.27(1.18)
Frontal Alpha 1	1.54(1.18)	1.39(1.34)	1.73(1.39)	1.31(0.84)	1.79(1.39)	1.47(1.19)	1.29(1.53)
Frontal Alpha 2	1.24(0.97)	1.07(0.77)	1.38(1.10)	1.07(0.77)	1.45(1.14)	1.19(0.75)	0.90(0.77)
Frontal Beta	0.94(0.85	0.57(0.40)	1.09(0.91)	0.74(0.74)	1.16(0.91)	0.64(0.42)	0.48(0.36)
Frontal Gamma 1	0.83(0.84)	0.44(0.31)	0.99(0.89)	0.64(0.73)	1.04(0.89)	0.48(0.30)	0.38(0.31)
Frontal Gamma 2	0.79(0.81)	0.42(0.29)	0.94(0.86)	0.61(0.72)	0.99(0.86)	0.45(0.29)	0.36(0.31)
Posterior Delta	3.86(3.84)	2.11(2.23)	4.85(4.52)	2.69(2.41)	5.02(4.66)	2.57(2.69)	1.49(1.17)
Posterior Theta	2.91(2.83)	1.47(1.57)	3.80(3.41)	1.85(1.38)	4.09(3.69)	1.75(1.88)	1.07(0.91)
Posterior Alpha 1	1.82(1.38)	1.57(1.71)	2.19(1.63)	1.39(0.85)	2.22(1.51)	1.94(2.07)	1.06(0.82)
Posterior Alpha 2	1.51(1.18)	1.23(0.98)	1.79(1.39)	1.16(0.73)	1.82(1.29)	1.52(1.13)	0.84(0.53)
Posterior Beta	1.00(1.00)	0.56(0.51)	1.26(1.19)	0.71(0.60)	1.24(1.02)	0.68(0.61)	0.40(0.24)
Posterior Gamma 1	0.90(0.97)	0.45(.0.44)	1.14(1.16)	0.62(0.60)	1.12(0.97)	0.54(0.54)	0.32(0.20)
Posterior Gamma 2	0.86(0.93)	0.43(0.44)	1.09(1.10)	0.59(0.59)	1.06(0.92)	0.52(0.53)	0.31(0.19)
Whole Head Delta	3.95(4.24)	2.07(1.82)	4.49(4.22)	3.29(4.24)	4.48(3.84)	2.47(2.13)	1.53(1.13)
Whole Head Theta	2.58(2.40)	1.24(1.21)	3.12(2.67)	1.94(1.89)	3.35(2.76)	1.42(1.35)	0.99(0.96)
Whole Head Alpha 1	1.13(0.81)	1.23(1.38)	1.24(0.94)	1.01(0.62)	1.23(0.76)	1.41(1.52)	0.99(1.15)
Whole Head Alpha 2	0.76(0.49)	0.87(0.67)	0.81(0.55)	0.71 (0.40)	0.81(0.47)	1.02(0.72)	0.66(0.54)
Whole Head Beta	0.30(0.23)	0.21(0.14)	0.36(0.27)	0.23(0.15)	0.35(0.19)	0.25(0.16)	0.16(0.08)
Whole Head Gamma 1	0.18(0.19)	0.08(0.04)	0.23(0.22)	0.12(0.12)	0.21(0.13)	0.09(0.05)	0.07(0.03)
Whole Head Gamma 2	0.13(0.13)	0.06(0.04)	0.16(0.16)	0.08(0.09)	0.15(0.08)	0.06(0.04)	0.05(0.03)

Note: Cell notation is mean (standard deviation). All values scaled to picovolts.

**Table 5.** Means for Relative Resting Power by Group and Sub-Group

Variable	All FXS	All CON	FXS males	FXS females	FXS non-mosaic males	CON males	CON females
**N**	70	71	38	32	27	41	30
Frontal Delta	0.40(0.11)	0.36(0.09)	0.41(0.09)	0.39(0.12)	0.40(0.09)	0.36(0.09)	0.35(0.11)
Frontal Theta	0.30(0.06)	0.24(0.05)	0.32(0.05)	0.29(0.07)	0.33(0.06)	0.24(0.05)	0.24(0.05)
Frontal Alpha 1	0.19(0.04)	0.22(0.06)	0.18(0.03)	0.19(0.05)	0.17(0.03)	0.22(0.05)	0.22(0.07)
Frontal Alpha 2	0.15(0.03)	0.19(0.04)	0.14(0.02)	0.16(0.03)	0.14(0.02)	0.19(0.04)	0.18(0.04)
Frontal Beta	0.10(0.02)	0.09(0.02)	0.10(0.02)	0.09(0.02)	0.11(0.02)	0.09(0.02)	0.09(0.02)
Frontal Gamma 1	0.08(0.02)	0.08(0.02)	0.09(0.02)	0.08(0.02)	0.09(0.02)	0.07(0.02)	0.08(0.02)
Frontal Gamma 2	0.08(0.02)	0.07(0.02)	0.08(0.02)	0.07(0.02)	0.08(0.02)	0.07(0.02)	0.07(0.02)
Posterior Delta	0.39(0.11)	0.33(0.11)	0.39(0.09)	0.39(0.13)	0.39(0.09)	0.32(0.11)	0.34(0.11)
Posterior Theta	0.29(0.06)	0.23(0.06)	0.31(0.05)	0.27(0.06)	0.32(0.05)	0.23(0.06)	0.24(0.05)
Posterior Alpha 1	0.20(0.04)	0.25(0.06)	0.19(0.03)	0.22(0.05)	0.19(0.03)	0.25(0.06)	0.24(0.05)
Posterior Alpha 2	0.17(0.03)	0.21(0.05)	0.16(0.02)	0.18(0.04)	0.15(0.02)	0.21(0.05)	0.20(0.05)
Posterior Beta	0.10(0.02)	0.09(0.02)	0.10(0.02)	0.10(0.02)	0.10(0.02)	0.09(0.02)	0.09(0.02)
Posterior Gamma 1	0.09(0.02)	0.07(0.02)	0.09(0.02)	0.08(0.03)	0.09(0.02)	0.07(0.02)	0.08(0.05)
Posterior Gamma 2	0.08(0.02)	0.07(0.02)	0.08(0.02)	0.08(0.03)	0.08(0.02)	0.07(0.02)	0.07(0.02)
Whole Head Delta	0.39(0.11)	0.35(0.09)	0.39(0.09)	0.38(0.13)	0.39(0.09)	0.35(0.09)	0.35(0.11)
Whole Head Theta	0.28(0.09)	0.21(0.05)	0.29(0.07)	0.26(0.09)	0.31(0.08)	0.20(0.06)	0.21(0.05)
Whole Head Alpha 1	0.15(0.06)	0.21(0.09)	0.14(0.04)	0.17(0.08)	0.13(0.04)	0.21(0.08)	0.20(0.09)
Whole Head Alpha 2	0.11(0.04)	0.16(0.07)	0.09(0.03)	0.12(0.05)	0.09(0.03)	0.17(0.06)	0.16(0.07)
Whole Head Beta	0.04(0.02)	0.04(0.02)	0.04(0.02)	0.04(0.01)	0.04(0.03)	0.04(0.02)	0.05(0.02)
Whole Head Gamma 1	0.02(0.01)	0.02(0.01)	0.02(0.01)	0.02(0.01)	0.02(0.01)	0.02(0.01)	0.02(0.01)
Whole Head Gamma 2	.02(0.01)	0.01(0.01)	0.02(0.01)	0.01(0.01)	0.02(0.01)	0.01(0.01)	0.02(0.01)

Note. Cell notation is mean (standard deviation). All values represent proportion of total power.

**Table 6.** Means for Resting Alpha Peak and Chirp by Group and Sub-Group

Variable	All FXS	All CON	FXS males	FXS females	FXS non-mosaic males	CON males	CON females
**RESTING ALPHA PEAK**							
**N**	70	71	38	32	27	41	30
Frontal	8.55(0.39)	8.46(0.33)	8.56(0.41)	8.54(0.39)	8.56(0.45)	8.49(0.34)	8.42(0.31)
Posterior	8.17(0.52)	8.57(0.29)	8.18(0.53)	8.16(0.51)	8.21(0.57)	8.59(0.28)	8.53(0.31)
Whole Head	8.35(0.27)	8.53(0.18)	8.31(0.22)	8.31(0.31)	8.32(0.22)	8.53(0.19)	8.54(0.17)
**CHIRP TASK**							
**N**	57	57	37	20	26	34	23
Gamma Power	7.07(1.80)	5.45(1.88)	7.65(1.79)	6.01(1.27)	8.09(1.78)	5.64(1.36)	5.21 (2.47)
Theta Power	24.25(2.94)	22.19(2.25)	24.14(2.85)	24.45(3.17)	24.39(2.87)	22.48(2.07)	21.76(2.47)
Alpha Power	−0.21(0.33)	−0.06(0.32)	−0.21 (0.36)	−0.21 (0.28)	−0.22(0.37)	−0.04(0.33)	−0.08(0.31)
Low Gamma ITPC	0.10(0.06)	0.14(0.08)	0.09(0.06)	0.13(0.06)	0.08(0.06)	0.15(0.08)	0.13(0.07)
High Gamma ITPC	0.04(0.03)	0.03(0.02)	0.03(0.03)	0.04(0.03)	0.04(0.04)	0.04(0.03)	0.03(0.02)
Stimulus Onset ITPC	0.14(0.08)	0.08(0.06)	0.14(0.08)	0.12(0.07)	0.14(0.08)	0.08(0.06)	0.08(0.06)
Stimulus Offset ITPC	0.05(0.04)	0.04(0.04)	0.06(0.04)	0.05(0.04)	0.06(0.04)	0.05(0.04)	0.04(0.05)

Note: Cell notation is mean (standard deviation). Alpha peak values are in Hertz. Power values are log scaled to microvolts squared. Gamma and theta power are absolute power. Alpha power is baseline corrected absolute power. Intertrial phase coherence (ITPC) scaled to critical R (range 0–1).

**Table 7.** Classification Results for Chirp

Variable	AUC – 30% holdout test set	AUC – entire dataset	Cross Validation Classification Error
**ALL SUBJECTS**			
Gamma Power	.7266	.7270	.3596
Theta Power	.7785	.7208	.3158
Alpha Power	.7093	.6174	.4825
Low Gamma ITPC	.6540	.6334	.4123
High Gamma ITPC	.5260	.5334	.4561
Stimulus Onset ITPC	.7093	.7153	.3509
Stimulus Offset ITPC	.5329	.6011	.4386
All ITPC Variables	.6159	.7532	.3158
All Power Variables	.8651	.7907	.2807
Gamma Power/Low Gamma ITPC	.7855	.7393	.3684
All Variables	.7855	.8495	.2982
**SUB-GROUP ANALYSES**			
**MALES ONLY**			
All Variables	.9364	.9118	.2676
All Power Variables	.9091	.9030	.2394
**FEMALES ONLY**			
All Variables	.5556	.8783	.3256
All Power Variables	.9444	.9522	.3256
**NON-MOSAIC MALES ONLY**			
All Variables	.9610	.9299	.2167
All Power Variables	.8875	.9355	.2333

Note: Top 2 highest performing variables for all subjects are highlighted in grey. Sub-group analyses for males, females, and non-mosaic males reflect these 2 variables only.

## Data Availability

EEG data are available in raw form on the NIMH Data Archive, collection ID 3766. Cleaned datasets used and/or analyzed during the current study are available from the corresponding author on reasonable request.
